# Comparative transcriptome analysis of nonchilled, chilled, and late-pink bud reveals flowering pathway genes involved in chilling-mediated flowering in blueberry

**DOI:** 10.1186/s12870-018-1311-8

**Published:** 2018-05-31

**Authors:** Guo-qing Song, Qiuxia Chen

**Affiliations:** Plant Biotechnology Resource and Outreach Center, Department of Horticulture, Michigan State University, East Lansing, MI 48824 USA

**Keywords:** Chilling requirement, Cold hardness, Flowering time control, Freezing tolerance, *Vaccinium corymbosum*, Vernalization, Woody plant

## Abstract

**Background:**

Blueberry cultivars require a fixed quantity of chilling hours during winter endo-dormancy for vernalization. In this study, transcriptome analysis using RNA sequencing data from nonchilled, chilled, and late pink buds of southern highbush blueberry ‘Legacy’ was performed to reveal genes associated with chilling accumulation and bud break.

**Results:**

Fully chilled ‘Legacy’ plants flowered normally whereas nonchilled plants could not flower. Compared to nonchilled flower buds, chilled flower buds showed differential expression of 89% of flowering pathway genes, 86% of MADS-box genes, and 84% of cold-regulated genes. Blueberry orthologues of *FLOWERING LOCUS T* (*FT*) did not show a differential expression in chilled flower buds (compared to nonchilled flower bud) but were up-regulated in late-pink buds (compared to chilled flower bud). Orthologoues of major MADS-box genes were significantly up-regulated in chilled flower buds and down-regulated in late-pink buds. Functional orthologues of *FLOWERING LOCUS C* (*FLC*) were not found in blueberry. Orthologues of *Protein FD (FD), TERMINAL FLOWER 1 (TFL1)*, and *LEAFY (LFY)* were down-regulated in chilled flower buds and in late-pink buds compared to nonchilled flower bud.

**Conclusions:**

The changes from nonchilled to chilled and chilled to late-pink buds are associated with transcriptional changes in a large number of differentially expressed (DE) phytohormone-related genes and DE flowering pathway genes. The profile of DE genes suggests that orthologues of *FT, FD*, *TFL1*, *LFY*, and MADS-box genes are the major genes involved in chilling-mediated blueberry bud-break. The results contribute to the comprehensive investigation of the vernalization-mediated flowering mechanism in woody plants.

**Electronic supplementary material:**

The online version of this article (10.1186/s12870-018-1311-8) contains supplementary material, which is available to authorized users.

## Background

Winter dormancy (endo-dormancy) is essential for deciduous fruit crop survival [1, 2]. Under inductive low temperatures in the fall, deciduous woody fruit and nut crops are acclimated to develop freezing tolerance; meanwhile, accumulation of effective chilling hours is stimulated [3]. Sufficient chilling accumulation gives plants full vernalization, which is a prerequisite for bud-break in the spring.

Climate change in the last 40 years has caused earlier shifts in the onset of the growing season for trees (e.g.*,* 2.3 days/decade in temperate Europe) and increased temperature fluctuation [4]. Early onset of the growing season causes insufficient chilling hours and prevents bud-break in fruit trees. Increased temperature fluctuation during plant bloom turns early season frosts into a danger, with freezing injuries to flowers and young fruits [5]. Plant breeding to manipulate chilling requirements and develop improved freeze tolerant cultivars are considered to be long-term solutions to mitigate reduced winter chill, decrease freezing damages, and secure deciduous fruit production [6].

Seasonal flowering plays a significant role in a plant’s life cycle and is controlled by a network of flowering pathway genes [7–9]. *FLOWERING LOCUS C* (*FLC*) is a key regulator in the vernalization pathway of winter-annual *Arabidopsis thaliana* ecotypes [7]. In winter wheat and barley, *VERNALIZATION2* (*VRN2*) is a major regulator of vernalization-mediated flowering [8]. *FLC* and *VRN2* analogs are recorded in peach (*Prunus persica*). These analogs are the *DORMANCY ASSOCIATED MADS-box* (*DAM*) genes which are a cluster of six MADS-box transcription factors. The loss of all or part of the *DAMs* resulted in the non-vernalized peach *evergrowing* mutant [10, 11]. The *DAM* genes are considered alternatives to *FLC* in regulating vernalization-mediated chilling requirement and flowering [10, 12]. However, the *DAM* genes show high similarities to *A. thaliana AGAMOUS-LIKE 24* (*AGL24*) and *SHORT VEGETATIVE PHASE* (*SVP*) genes [12, 13]. Additionally, functional analysis of *DAMs* to reveal their roles in chilling-mediated flowering through reverse genetics has not been reported in peach. To date, neither a functional *FLC-LIKE* nor a *VRN2-LIKE* gene has been verified in *Vaccinium* plants [14].

Blueberries and cranberries are the most important *Vaccinium* fruits due to their high antioxidant and anti-inflammatory capacities [15]. Deciphering the mechanism of vernalization/chilling-mediated flowering will facilitate molecular breeding of blueberry cultivars for low-chilling requirement. To investigate flowering responses under nonconducive conditions, functional analysis of a blueberry *FLOWERING LOCUS T* (*VcFT*) gene has been conducted in highbush blueberry (*Vaccinium corymbosum* L.) [16–18]. Overexpression of *VcFT* (about 2900-fold increase in leaf tissues) caused continuous and precocious flowering in in vitro shoots and in one-year old ‘Aurora’ plants [16]. However, Overexpression of *VcFT* was not capable of fulfilling all chilling requirements in blueberry [17, 18]. Over 80% of the flower buds in two- and three-year old *VcFT*-overexpressing plants could not bloom under greenhouse conditions without a chilling period. To discover vernalization/chilling-responsive genes, transcriptome analyses were conducted with blueberry flower buds. The profile of differentially expressed (DE) genes will facilitate our understanding of the role of vernalization/chilling in blueberry bud-break.

## Results

### Identification of DE transcripts in chilled flower buds

**Fig. 1:**
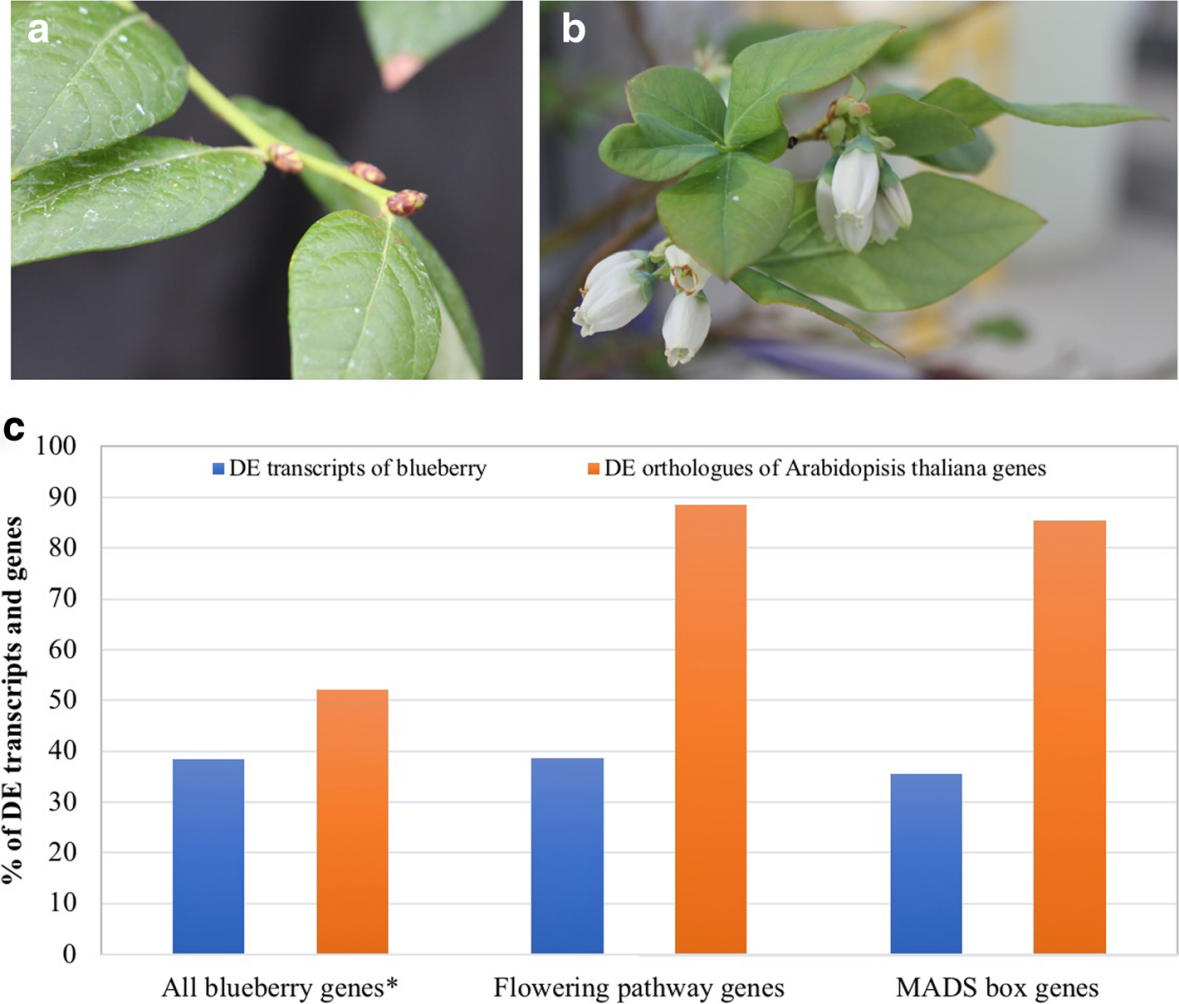
Effect of vernalization of blueberry flowering. **a** Nonchilled flower buds could not bloom. **b** Early bloom of fully chilled flower buds. **c** Percentages of differentially expressed (DE) genes and transcripts in chilled flower buds (in comparison to nonchilled flower buds). *DE genes including *A. thaliana* genes and genes annotated to other species

Nonchilled flower buds from the southern highbush blueberry ‘Legacy’ were grown in a heated greenhouse through the winter season. These buds did not flower in the following spring. In contrast, flower buds fully chilled under natural winter conditions flowered normally (Fig. 1). Transcriptome comparison of the chilled to nonchilled flower buds using Trinity and a non-annotated transcriptome reference (Reftrinity) of tetraploid blueberry in GenBank (Accession number: SRX2728597) [19, 20] revealed 37,000 differentially expressed genes (DEGs) and 47,000 DE transcripts.

To conduct transcriptome analysis, Reftrinity (180,000 genes and 250,000 isoforms) was annotated using Trinotate [20]. The annotation resulted in 14,000 known genes from 30,000 genes and 55,000 isoforms of Reftrinity. With this annotated reference, 64% of known blueberry genes and isoforms showed differential expression in the comparison of chilled to nonchilled flower buds (herein referring to DEGs/DE transcripts in chilled flower buds). Chilling affected expression of numerous genes simultaneously in blueberry flower buds.

### Effect of chilling accumulation on flowering pathway genes

Differential expression was detected in chilled blueberry flower buds for 89% of flowering pathway orthologues of known *A. thaliana* flowering pathway genes (Table 1; Fig. 1). One of the top two orthologues of *FT* (*VcFT*: hereafter *Vc* before any *A. thaliana* gene refers to the blueberry orthologue gene) showed a slight decrease while the other showed a slight increase. Similar results were observed for *CONSTANS-LIKE2* orthologues (Table 1).

**Table 1:** Differentially expressed major flowering pathway genes in both chilled flower buds (CB) [vs nonchilled flower buds (NB)] and late-pink buds (LPB) (vs CB) in ‘Legacy’. LogFC for chilled buds: Log_2_(CB/NB). LogFC for late-pink buds: Log_2_(LPB/CB)

Arabidopsis_ID|Vc_transcript_ID	LogFC for chilled buds	LogFC for late-pink buds	FDR for chilled buds	FDR for late-pink buds	e-value_to_blast	Gene_name	Annotation_by_Trinotate	Query_PmDAMs
AT5G60910.1|c77424_g2_i2	1.0	2.2	6E-03	3E-19	9E-44	FUL, AGL8	AGL11_ARATH	
AT1G69120.1|c92021_g1_i1	−0.6	−1.8	4E-02	5E-15	1E-80	AP1, AGL7	AGL8_SOLTU	
AT5G60910.1|c88116_g1_i1	−0.4	0.8	4E-02	1E-10	5E-91	FUL, AGL8	AGL8_SOLTU	
AT1G69120.1|c88116_g8_i1	3.2	−5.4	1E-16	2E-59	4E-94	AP1, AGL7	AGL8_SOLTU	
AT1G69120.1|c81830_g1_i1	3.5	−2.1	2E-42	4E-24	2E-52	AP1, AGL7	AGL9_PETHY	
AT1G69120.1|c88116_g7_i1	3.6	−2.7	3E-33	7E-41	3E-90	AP1, AGL7	AP1_SINAL	
AT5G15840.1|c77980_g1_i1	−0.5	0.7	2E-03	3E-10	1E-66	CO, FG	COL2_ARATH	
AT5G15840.1|c76265_g1_i1	2.3	−2.5	2E-09	6E-14	4E-30	CO, FG	COL2_ARATH	
AT5G60910.1|c79125_g1_i1	−0.3	−1.1	4E-02	2E-13	2E-35	FUL, AGL8	DEFA_ANTMA	
AT5G60910.1|c67980_g1_i1	3.4	−4.6	7E-46	2E-70	5E-36	FUL, AGL8	DEFA_ANTMA	
AT5G61850.1|c96427_g2_i1	−2.4	−5.7	4E-13	4E-24	7E-102	LFY, LFY3	FLO_ANTMA	
AT5G61850.1|c96427_g2_i2	−2.1	−6.7	2E-13	9E-31	3E-98	LFY, LFY3	FLO_ANTMA	
AT1G65480.1|c84088_g2_i3	−0.9	−4.5	2E-02	7E-12	5E-95	FT	HD3A_ORYSJ	
AT1G65480.1|c84088_g2_i5	1.1	−4.9	1E-03	9E-28	1E-94	FT	HD3A_ORYSJ	
AT1G26310.1|c77146_g1_i1	4.9	−3.1	2E-57	9E-37	2E-56	CAL, AGL10	MADS6_ORYSJ	
AT2G45660.1|c89673_g3_i1	2.6	−4.0	1E-09	3E-15	3E-53	SOC1, AGL20	SOC1_ARATH	
AT2G45660.1|c86010_g2_i1	3.9	−4.4	1E-08	2E-09	2E-54	SOC1, AGL20	SOC1_ARATH	
AT2G22540.1|c91377_g1_i7	1.0	−1.6	3E-02	1E-03	5E-69	SVP, AGL22	SVP_ARATH	
AT2G22540.1|c90829_g2_i1	1.2	−1.2	1E-03	5E-04	4E-77	SVP, AGL22	SVP_ARATH	
AT4G24540.1|c91377_g1_i7	1.0	−1.6	3E-02	1E-03	1E-39	AGL24	SVP_ARATH	
AT5G10140.1|c77146_g1_i1	4.9	−3.1	2E-57	9E-37	2E-31	FLC, FLF, AGL25	MADS6_ORYSJ	
AT5G10140.1|c88116_g6_i1	4.1	−1.9	5E-91	1E-26	7E-31	FLC, FLF, AGL25	SEP1_ARATH	
AT4G18960.1|c77424_g2_i2	1.0	2.2	6E-03	3E-19	1E-72	MAF2, AGL31	AGL11_ARATH	PmDAM1
AT2G14210.1|c80388_g1_i2	3.9	3.2	4E-02	5E-09	3E-79	MAF2, AGL31	MAD23_ORYSJ	PmDAM1
AT5G60910.1|c77146_g1_i1	4.9	−3.1	2E-57	9E-37	7E-54	MAF2, AGL31	MADS6_ORYSJ	PmDAM1
AT4G22950.1|c77424_g2_i2	1.0	2.2	6E-03	3E-19	1E-42	MAF3, AGL70	AGL11_ARATH	PmDAM1
AT4G18960.1|c88293_g3_i1	4.7	−4.7	5E-21	5E-20	5E-59	MAF4	AG_TOBAC	
AT5G15800.2|c88116_g6_i1	4.1	−1.9	5E-91	1E-26	2E-87	MAF4	SEP1_ARATH	PmDAM1
AT1G24260.2|c68983_g1_i1	2.5	0.0	3E-06	0E + 00	3E-42	MAF5, AGL68	.	
AT5G15800.2|c81830_g1_i1	3.5	−2.1	2E-42	4E-24	3E-89	MAF5, AGL68	AGL9_PETHY	PmDAM1

#N/A: No differential expression

Orthologues of seven major MADS-box genes in flowering pathway genes of blueberry buds, *APETALA1 (AP1)*, *FRUITFULL (FUL)*, *SUPPRESSOR OF OVEREXPRESSION OF CONSTANS 1 (SOC1)*, *CAULIFLOWER (CAL)*, *FLC, AGL24*, and *SVP*, were significantly up-regulated (Table 1). The orthologues of *Protein FD (FD), TERMINAL FLOWER 1 (TFL1)*, and *LEAFY (LFY),* and *ACTIN-RELATED PROTEIN6* (*ARP6*) were down-regulated genes. Both *VcFLC* and *VcSVP* showed a contrasting response of *FLC* to vernalization in *A. thaliana*. The down-regulated *VcFD* and up-regulated *VcSVP* support the DE *VcFT* results [7]. However, decreased expression of *VcLFY* contradicts the decreased *VcTFL1* and increased *VcAP1, VcAGL24*, and *VcSOC1* expressions. The unchanged expression of *VcFT* and decreased expression of *VcLFY* orthologues could prevent the flowering of chilled flower buds during plant vernalization.

The MADS-box transcription factor and flowering repressor, *FLC,* and *FRIGIDA* (*FRI*) are the major genes regulating the vernalization of *A. thaliana. FRI* activates *FLC,* but vernalization represses *FLC* [7]. Both *VcFRI* and *VcFLC* in blueberry are present in the annotated Reftrinity. *VcFRI* showed a high similarity to *FRI* (e = − 98) while two *VcFLC* transcripts showed a lower similarity to *FLC* (e = − 31). These two *VcFLC* transcripts were annotated to *SEP1* of *A. thaliana* and *MADS6* of rice, respectively (Table 1). In chilled flower buds, *VcFRI* did not show differential expression whereas expression of *VcFLC* was increased about 25-fold for the top-two *VcFLC* candidate genes (Table 1). When all 26 potential *VcFLC* candidate genes (e < − 20) were included in the analysis, four candidates showed a down-regulation with a fold-change of 0.6–0.8. The other 22 candidate genes showed an up-regulation with an average of 4.9-fold (Additional file 1: Table S2). The inconsistency between *VcFLC* and *FLC* response to chilling/vernalization suggests that a different or more complicated mechanism is involved in vernalization-mediated flowering in tetraploid, blueberry plants. Additionally, all other DEGs involved in known vernalization-mediated flowering pathway were up-regulated in chilled flower buds with the exception of *VcARP6* (c49456_g2, Log_2_FC = − 7.7) (Additional file 2: Table S1).

### Identification of DE transcripts in late-pink buds

Fully chilled blueberry flower buds remained dormant under chilling conditions in January until continuous warm conditions in April drove dormancy release and bloom. To investigate the effect of dormancy release on gene expression, RNA sequencing data was obtained from late-pink buds. Comparative transcripts analysis in late-pink buds to those in chilled flower buds (hereafter referring to DEGs/DE transcripts in late-pink buds) resulted in 28,000 DE isoforms, which were annotated to 11,000 known genes.

### Expression of flowering pathway genes in late-pink buds

The major DE flower pathway genes in chilled flower buds were compared to those in late-pink buds (Additional file 2: Table S1). The major flowering pathway genes *VcFT*, *VcSOC1*, *VcAP1*, *VcFUL*, *VcFLC*, *VcSVP*, and *VcLFY* showed decreased expression in late-pink buds. In *A. thaliana*, *FT*, *SOC1*, *AP1*, *FUL*, and *LFY* promote flowering while *SVP* and *FLC* are flowering repressors [7, 21]. The expression changes of *VcFD* and *VcTFL1* in late-pink buds were similar to those in chilled flower buds (Table 1). The decreased *VcFD* expression was related, at least in part, to *VcFT* down-regulation. Decreased *VcTFL1* expression in late-pink buds was associated with a down-regulation of *VcAP1* and *VcLFY.* Additionally, decreased *VcSVP* and *VcFLC* expression were associated with a decrease in *VcFT* expression.

### Expression of MADS-box genes

Blueberry DE MADS-box and *DAMs* genes were identified using *A. thaliana* MADS-box genes and Japanese apricot (*Prunus mume*) *DAMs* (*PmDAMs*) (Additional file 1: Table S2; Table 1). Orthologues of 62 *A. thaliana* MADS-box gene were identified in blueberry (unpublished data). DE orthologues of 53 and 44 MADS-box genes were detected in chilled flower buds and late-pink buds, respectively. These orthologues include the major flowering pathway genes *VcFLC*, *VcSOC1*, *VcSVP, VcAP1*, *VcFUL*, *VcCAL*, and *VcAGL24* (Table 1). The annotated *VcSOC1* (c86010_g2_i1) showed high similarities to 25 *A. thaliana* MADS-box genes. Similarly, the annotated *VcFLC* homologues were similar to 23 *A. thaliana* MADS-box genes (Table 1). The results suggest that *VcSOC1* and *VcFLC* could have multiple functions in blueberry.

Three DE *PmDAM* orthologues (*VcPmDAM1, VcPmDAM2,* and *VcPmDAM5)* were identified and showed high similarities to four *A. thaliana* MADS-box genes, *MADS AFFECTING FLOWERING 2* (*MAF2*), *MAF4*, *MAF5* and *FOREVER YOUNG FLOWER*. In *A. thaliana, MAF2*, *MAF4*, and *MAF5* are *FLC* paralogs. *MAF2*, *MAF5* and *FLC* are down-regulated and *MAF5* is up-regulated during vernalization [22]. In contrast, the blueberry orthologues *VcFLC, VcMAF1, VcMAF2, VcMAF4,* and *VcMAF5* were up-regulated while *VcMAF3* was repressed in chilled flower buds (Table 1; Additional file 1: Table S2). Additionally, three DE *VcPmDAMs* were annotated to the homologues *VcSOC1*, *VcSVP, VcAP1*, and *VcSEP1*. The up-regulated *VcPmDAM1* homologues were the only DE orthologue in chilled flower buds. In late-pink buds, 75% of DE *VcPmDAM1* homologues and all DE *VcPmDAM5* homologues were down-regulated while DE *VcPmDAM2* was up-regulated (Additional file 1: Table S2). These blueberry MADS-box genes showed significant changes in response to both chilling and flower bud breaking (Table 1). However, during vernalization, the responses of *VcFLC*, *VcMAF*s, and *VcPmDAMs* diverges from *FLC’s* response to vernalization in *A. thaliana*.

### Response of phytohormone-related genes in chilled and late-pink buds

**Fig. 2:**
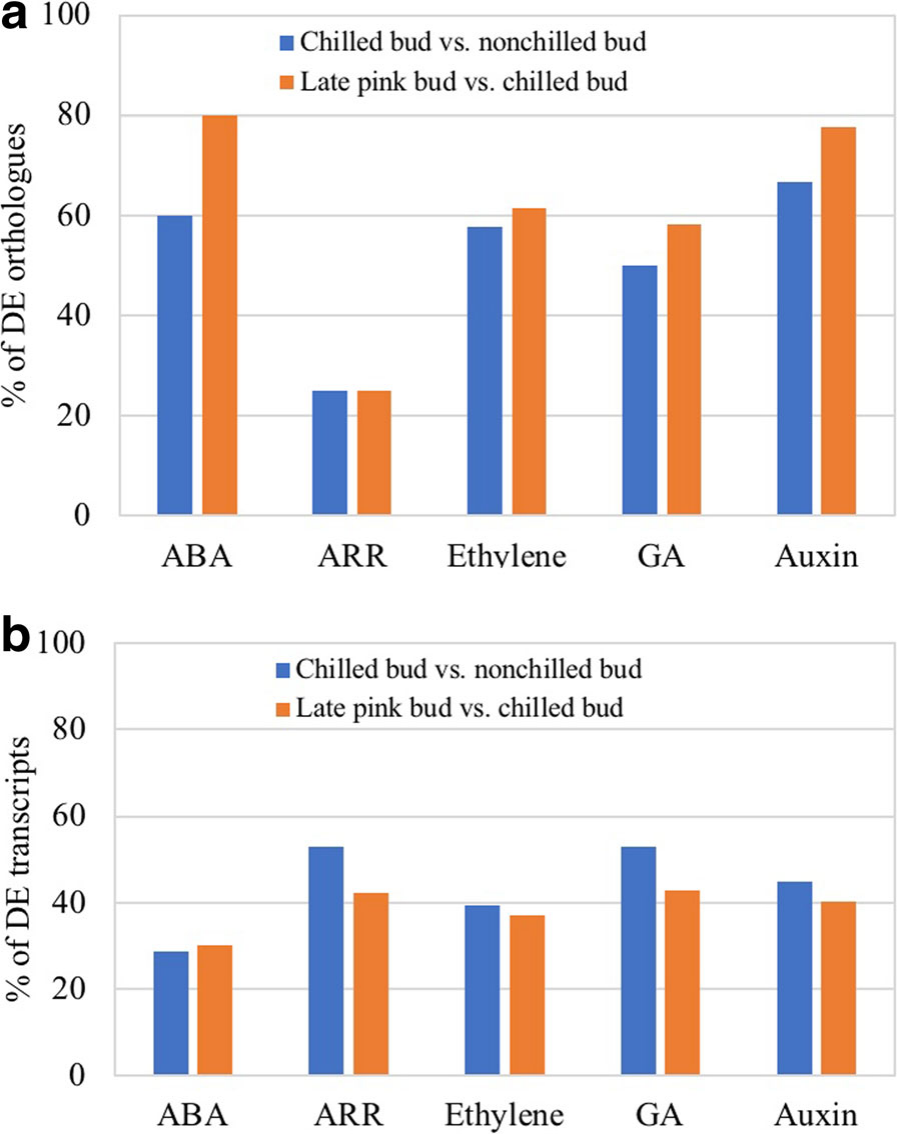
Response of phytohormone-related genes and transcripts in chilled flower buds (vs. nonchilled flower buds) and late-pink buds (vs. chilled flower buds). **a** Percentage of differentially expressed (DE) orthologues of *A. thaliana* genes (The number of DE *A. thaliana* genes ÷ total number of *A. thaliana* genes × 100). **b** Percentage of DE transcripts of transcripts of blueberry phytohormone-related genes (The number of DE transcripts ÷ total number of transcripts × 100)

For both chilled buds and late-pink buds, DE transcripts showed high similarities to the pathway genes for five major phytohormones (Additional file 3: Table S3). Over 50% of the DE blueberry orthologues were related to abscisic acid (ABA), ethylene, auxin, and gibberellin (GA) genes while 25% were related to cytokinin genes (Fig. 2). The late-pink bud showed more DE phyotohormone orthologues than the chilled buds (Fig. 2). The DE phytohormone genes suggest the potential involvement of these phytohormones during chilling and flowering.

**Fig. 3:**
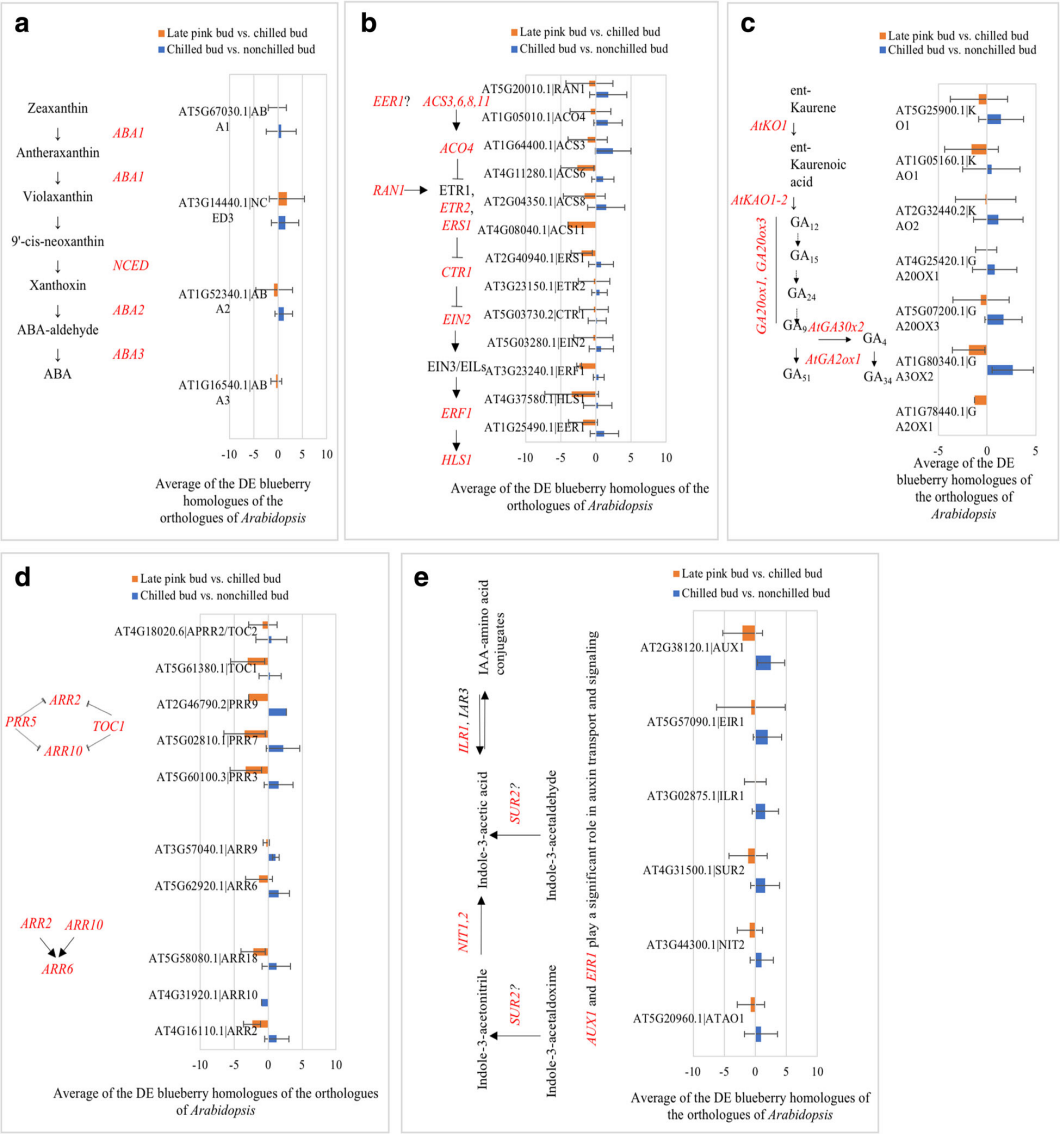
Average fold changes (Log_2_FC) of differentially expressed homologues for each of the phytohormone-related orthologue of *A. thaliana* in chilled flower buds (CB) [vs. nonchilled flower buds (NB)] and late-pink buds (LPB) [vs. chilled flower buds (CB)]. LogFC for chilled buds: Log_2_(CB/NB). LogFC for late-pink buds: Log_2_(LPB/CB). **a** Abscisic acid biosynthesis pathway genes [46]. **b** Ethylene biosynthesis and signaling pathway genes [45, 47]. **c** Gibberellin biosynthesis pathway genes [48]. **d** Two-component *ARABIDOPSIS RESPONSE REGULATORS* (ARR) [49, 50]. **e** Auxin biosynthesis pathway genes [51]. The bars represent standard deviation

Due to the tetraploid nature of ‘Legacy’, orthologues of each *A. thaliana* gene used for query often have more than one homologue (Fig. 2). Thus, an average of the changes (Log_2_^Fold Change^) for all the DE transcripts that are orthologues derived from a single *A. thaliana* query gene was used to represent the overall change of each phytohormone-related gene (Fig. 3). Increased expression of *ABA1*, *ABA2* and *NINE-CIS-EPOXYCAROTENOID DIOXYGENASE 3* (*NCED3*) in the ABA biosynthesis pathway were seen in chilled flower buds. *ABA1* and *ABA2* continued to increase and *NCED3* decreased in late-pink buds (Fig. 3). The increased expression of these orthologues indicates a potential increase in ABA biosynthesis during vernalization. Regardless of decreased *ABA1* expression, increased *NCED3* expression suggests that there is an increase in ABA biosynthesis during floral bud break (Fig. 3).

DE orthologues of ethylene signaling pathway genes were all up-regulated in chilled buds and down-regulated in flowering buds (Fig. 3). These orthologues are considered regulators for freezing tolerance in *A. thaliana*. Indole-3-acetic acid (IAA) and GA biosynthesis pathway orthologues were up-regulated in chilled flower buds and decreased in late-pink buds (Fig. 3). *GIBBERELLIN 3-BETA-DIOXYGENASE 2* (*GA3OX2*) in the GA pathway and *AUX1* in the IAA pathway were major DEGs with high expression changes. The DE *ARABIDOPSIS RESPONSE REGULATORS* **(***ARRs*) orthologues included two A-type, two B-type and five ARR-like genes in chilled buds and flowering buds. One B-type orthologue (*ARR10*) was suppressed only in chilled buds (Fig. 3).

### Gene networks of DEGs in chilled flower buds and late-pink buds

**Fig. 4:**
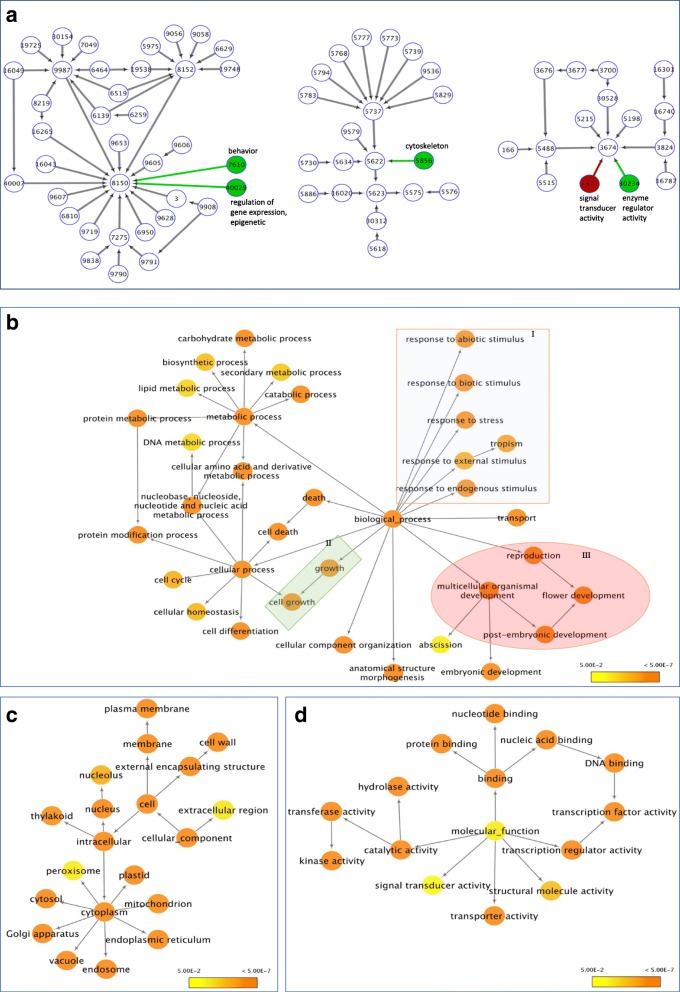
Gene networks of differentially expressed genes (DEGs) in chilled flower buds and late-pink buds. DEGs in chilled flower buds were identified in comparison to nonchilled flower buds while DEGs in late-pink buds were identified in comparison to chilled flower buds. The ontology file of GOSlim_Plants in BiNGO was used to identify over-represented GO terms (*P* < 0.05). **a** Comparison of the gene network in chilled flower buds to late-pink buds; white nodes and black edges are present in both gene networks; red nodes and edges are present only in the chilled buds; and green nodes and edges are present only in the late-pink buds. The number in each circle is a GO identity number. A gene network in chilled flower buds (**b** “Biological_process” **c** “Cellular component” **d** “Molecular function”). I, II, and III in **b** show GO terms related to stress, plant growth, and reproduction, respectively

Over-represented Gene Ontology (GO) terms (*P* < 0.05) were grouped to visualize gene networks of the annotated DE transcripts using the GOslim_Plant as the selected GO file and *A. thaliana* annotation as the reference. The DE transcripts were classified in 70 and 73 over-represented GO terms for chilled flower buds and late-pink buds, respectively (Fig. 4). The over-represented GO terms for chilled flower buds and late-pink buds were identical except for two GO terms (Fig. 4), suggesting that the same transcripts responded to temperature changes in these buds. The difference in “biological_process” was two additional over-represented GO terms (GO:0007610-behavior and GO:040029-regulation of gene expression, epigenetic) found in late-pink buds but not in chilled flower buds. (Fig. 4).

Over-represented GO terms in chilled flower buds revealed the impact of chilling on GO terms in three categories (Fig. 4). The over-represented GO terms in “biological_process” revealed that vernalization/chilling affected expression of genes in multiple GO terms related to growth, response to stress, and reproduction (Fig. 4). The gene network based on over-represented GO terms facilitate our understanding of the role DEGs in both chilled flower buds and late-pink buds (Fig. 4).

### Validation of the expression of selected genes

**Fig. 5:**
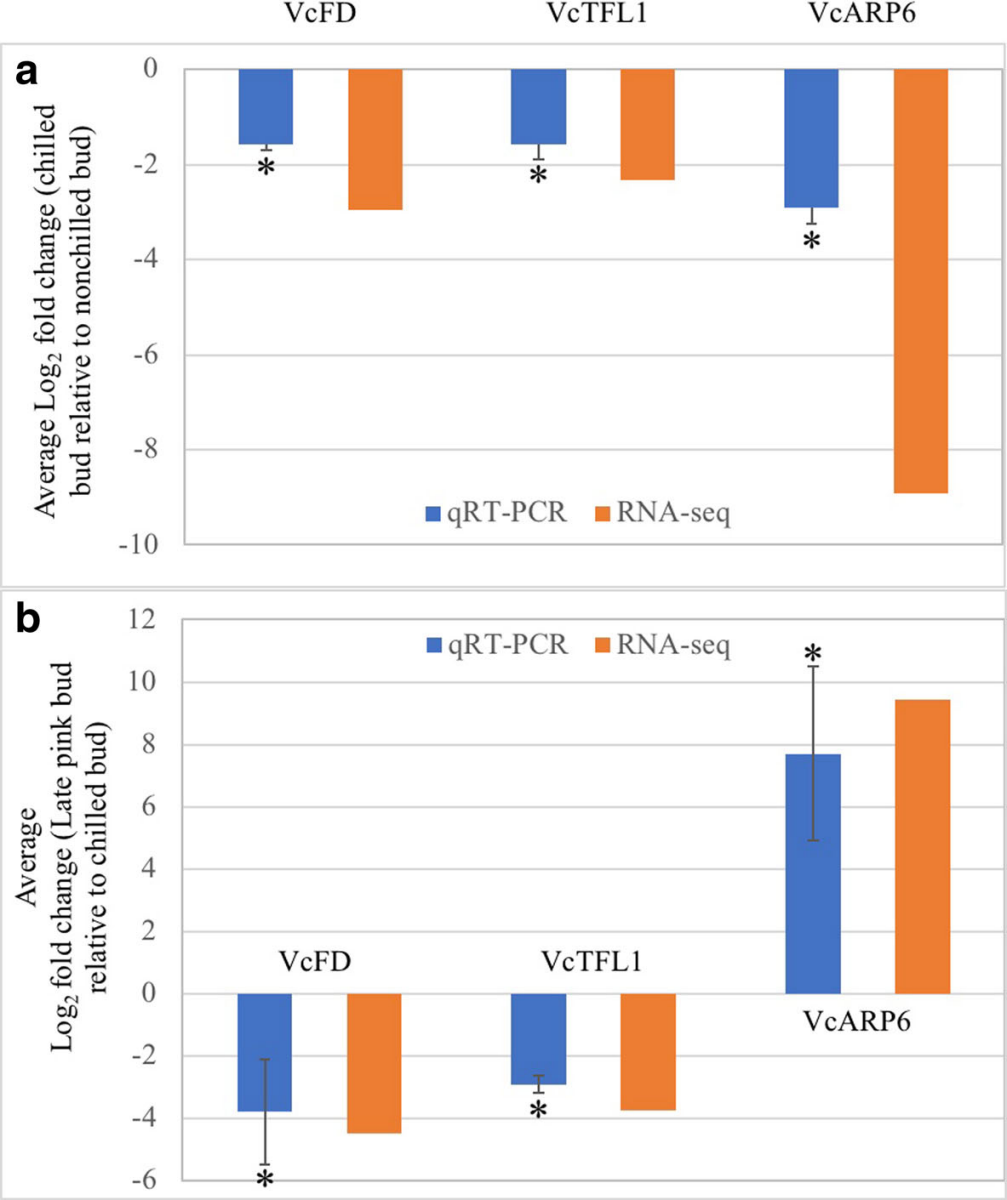
Comparison of RNA sequencing and qRT-PCR analysis of three differentially expressed genes in (**a**) chilled flower buds (compared to nonchilled flower buds) (**b**) and late-pink buds (compared to chilled flower buds). Eukaryotic translation initiation factor 3 subunit H is the internal control. Log_2_fold-change was calculated by -∆∆Ct = − [(Ct_GOI_ – Ct_nom_) _tissue 1_ – (Ct_GOI_ – Ct_nom_) _tissue 2_]. Average Log_2_fold-change ± standard deviation of three biological replicates. Significant average fold-change determined using a Student’s *t*-test is denoted. An asterisk (*) indicates *p* < 0.001

In chilled flower buds and late-pink buds, qRT-PCR were used to validate DE transcripts of *VcFD*, *VcTFL1*, and *VcARP6* (Fig. 5). The results suggested high-reliability of the RNA-seq data.

## Discussion

Transcriptome analysis is an effective approach to study flowering pathway genes [23, 24]. Using this approach, DEGs in response to vernalization have been identified in Japanese pear (*Pyrus pyrifolia* Nakai) and oriental lily [24]. For blueberries, expressed sequence tags have been generated from blueberry flower buds [25]. However, comparative transcriptome analyses of different stage floral buds have not been documented.

### The roles of *VcFT*, *VcLFY* and *VcARP6* in vernalization-mediated blueberry flowering

**Fig. 6:**
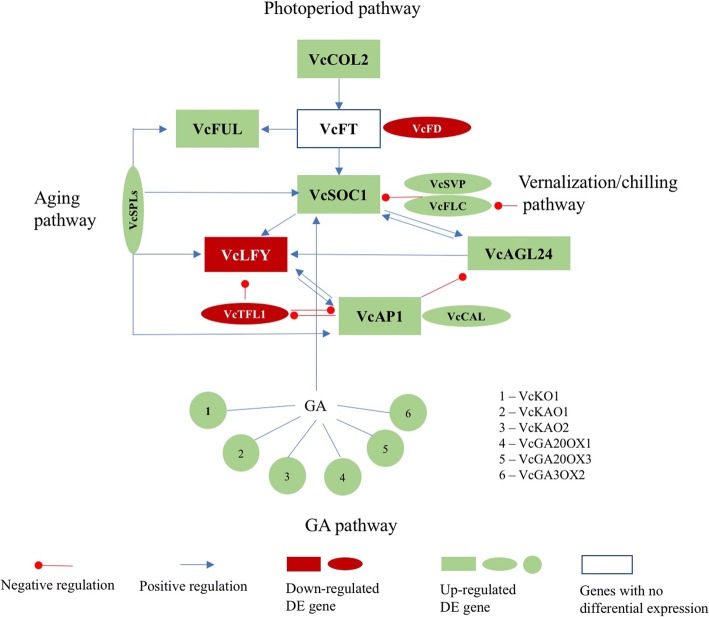
Response of major flowering pathway genes in chilled flower buds (compared to nonchilled flower buds). The relationships among the listed genes are drawn according to the diagram for *A. thaliana* by Fornara et al. 2010 [7], although not all DE genes of blueberry align perfectly with the correlations proposed for *A. thaliana*. All the listed genes in this diagram showed down-regulation in late-pink buds (compared to chilled flower buds) (Additional file 2: Table S1)

Overexpression of *VcFT* (expression level > 2000-fold in leaf tissues) resulted in precocious flowering [16, 17]. However, the high expression level did not completely reverse the need for chilling for normal plant flowering which suggests that chilling requirement is not replaceable by *VcFT* manipulation. When the results were aligned to the flowering pathway of *A. thaliana* [7], the acquired chill did not change *VcFT* expression in blueberry flower buds (Fig. 6). This result was also observed in woody pear but not in herbaceous lily [24]. The inactive *VcFT* expression in response to chilling may be one major reason that chilled flower buds remain dormant prior to exposure to bud-breaking temperatures since *VcFT* increased in late-pink buds (Table 1).

Overexpression of *VcFT* in leaves promoted expression of downstream genes *VcSOC1*, *VcFUL*, *VcAP1*, and *VcLFY* [17]. In this study, expression of *VcSOC1*, *VcFUL*, and *VcAP1* were up-regulated but *VcLFY* was repressed regardless of *VcFT* expression in flower buds (Table 1: Fig. 5). The expression of *VcFT*-downstream genes was regulated independently of *VcFT* in chilled flower buds. Additionally, repressed *VcLFY* response in chilled flower buds is similar to results observed in grapevine (*Vitis vinifera*) [26]. These results suggest that *VcLFY* supression may play a role in chilling-mediated flowering by maintaining bud dormancy before bud break.

The interaction of *FD* and *FT* promotes flowering in *A. thaliana* while *TFL1* is a negative regulator of *FT* [27, 28]. In this study, reduced *VcFD* and *VcTFL1* expressions did not changed *VcFT* expression in chilled flower buds. However, increased *VcFD* and decreased *VcTFL1* in late-pink buds were associated with increased *VcFT*. *TFL1* was considered a repressor of both *LFY* and *AP1* in *A. thaliana* until recent evidence suggest that *TFL1* transcription was suppressed by *AP1* but promoted by *LFY* [29, 30]. For chilled blueberry flower buds, increased expression of *VcAP1* and decreased expression of *VcLFY* were associated with decreased expression of *VcTFL1*. This result is consistent with the recent report about the interactions among these three genes [30]. During flower bud break, repressed *VcTFL1* associated with decreased *VcLFY* and *VcAP1* supports the theory that *TFL1* represses *LFY* [30]. Although some DEGs of flowering pathway genes in blueberry match the proposed interactions in *A. thaliana* [7], *VcFD* and *VcTFL1* seem to be playing a more significant role in blueberry (Fig. 4).

*FLC* interacts with *SVP* and both are repressed during vernalization in *A. thaliana* [19]. In chilled blueberry flower buds, both *VcFLC* and *VcSVP* homologues showed decreased expression but increased in late-pink buds (Table 1). In *A. thaliana*, *ARP6* activates *FLC*, *MAF4*, and *MAF5*, which are all repressors of plant flowering in vernalization pathway [31]. In blueberry, decreased *VcARP6* in chilled flower buds was not associated with decreased expression of *VcFLC*, *VcMAF4*, or *VcMAF5*. However, increased *VcARP*6 (c49456_g2, Log_2_FC = 8.1) was associated with an increase in these genes in late-pink buds (Table 1; Additional file 2: Table S1). Due to *ARP6*’s role in *A. thaliana* vernalization, DE *VcARP6* may significantly contribute to chilling-mediated flowering of blueberry flower buds.

### Expression of blueberry MADS-box genes in chilled flower buds and late-pink buds

The major flowering pathway genes *SOC1*, *FLC*, *AP1*, *FUL*, *SVP*, and *AGL24* are MADS-box genes encoding MIKC^c^ (classical MIKC) proteins [7]. Similar to *A. thaliana*, multiple blueberry MADS-box genes are present and activated at different flowering stages. *VcSOC1, VCAP1* and *VcFUL* are responsive to *VcFT* overexpression [17]. Additionally, constitutively expressed *VcSOC1* or Keratin-like (K) domain of *VcSOC1* promoted blueberry flowering [32]. In this study, the functional orthologues of *FLC* and *AGL24* were not detected in blueberry, suggesting that the vernalization/chilling-mediated flowering pathway of blueberry is different from *A. thaliana. VcSVP* showed differential expression in chilled and late-pink buds (Table 1).

In woody fruit crops, functional *FLC* orthologues have not been identified. The peach *DAM* genes mimic *FLC* response in *A. thaliana* under dormancy [10]. The *DAM* genes are the orthologues of *A. thaliana AGL24* and *SVP* genes [12, 33]. In this study, the DE *DAMs* showed similarity to several MADS-box genes (*VcAP1*, *VcSVP*, *VcSOC1*, and *VcSPL3*) (Table 1). Therefore, it is possible that the interaction of multiple MADS-box genes co-regulates chilling-mediated flowering in blueberry as well as other woody plants.

### Response of phytohormone genes during vernalization and devernalization

Phytohormones are involved in plant flowering and dormancy. In *A. thaliana*, cold acclimation, dormancy, and plant flowering are affected by phytohormone gene expression [7, 34, 35]. The gibberellin pathway interacts with the flowering pathway through *SOC1* [7, 36]. Ethylene signaling pathway genes are considered regulators for freezing tolerance in *A. thaliana*. In this study, DE phytohormone genes were identified in both chilled buds and late-pink buds of blueberry (Fig. 2; Additional file 3: Table S3). These DE phytohormone genes reveal their potential roles in cold acclimation, dormancy, freezing tolerance, and chilling-mediated flowering in blueberries. For example, chilled blueberry buds showed higher freezing tolerance than nonchilled buds and flower tissue [37]. Increased DE orthologues of ethylene genes in the chilled blueberry buds were responsible for the enhanced freezing tolerance in chilled buds while decreased expression of DE ethylene orthologues in late-pink buds reduced freezing tolerance (Fig. 3).

## Conclusions

The changes from nonchilled to chilled and chilled to late-pink buds are associated with transcriptional changes in a large number of DE phytohormone-related genes and DE flowering pathway genes. The DE flowering pathway genes suggest that orthologues of *FT, FD*, *TFL1*, *LFY*, and MADS-box genes are the major genes involved in chilling-mediated blueberry bud-break. The DE phytohormone genes reveal the potential roles of phytohormone genes in cold acclimation, dormancy, freezing tolerance, and chilling-mediated flowering in blueberries. The results contribute to the comprehensive investigation of the chilling-mediated flowering mechanism in woody plants.

## Method

### Plant materials

The tetraploid southern highbush blueberry ‘Legacy’ needs over 800 chilling units (CU) for normal flowering. Twelve 4-year old ‘Legacy’ plants were obtained through micropropagation of in vitro cultured shoots. All plants were grown in 4-gal pots in a secured courtyard under natural light conditions at Michigan State University, East Lansing, Michigan (latitude 42.701847, longitude − 84.482170). The average low and high temperatures in January 2016 were − 11 °C and − 1.8 °C, respectively (http://www.usclimatedata.com/climate/east-lansing/michigan/united-states/usmi0248). In September 2015, six plants were moved to a heated greenhouse with a 12-h photoperiod and a minimum temperature of 23 °C in order to keep the plants from any chilling hour accumulation. The remaining six plants were kept in the secured courtyard. In November, three plants were selected from the greenhouse and 30–50 flower buds were harvested per plant. These flower buds did not receive any chilling temperatures and were labeled as nonchilled flower buds. At the end of January 2016, three plants were selected from the courtyard and 30–50 flower buds were harvested per plants. These flower buds experienced natural chilling conditions through mid-winter and were labeled as chilled flower buds. In April, 20–30 flower buds per plant were obtained from a second harvest of the same three plants in the courtyard. These flower buds experienced natural chilling conditions and began to flower in early spring. The buds selected were at early-pink-stage and were labeled as late-pink buds. All tissues collected were frozen immediately in liquid nitrogen and stored at − 80 **°**C. Three plants for each bud type were used as the three biological replicates for transcriptome analysis.

### RNA preparation, sequencing, and de novo transcriptome assembly

Total RNA of each blueberry sample (from individual plants) was isolated from 200 mg of bud tissues using a separate CTAB method [38] and was purified using RNeasy Mini Kit (Qiagen, Valencia, CA, USA). All RNA samples were purified using On-Column DNase digestion with the RNase-free DNase Set (Qiagen). The integrity of the RNA samples was assessed using the Agilent RNA 6000 Pico Kit (Agilent Technologies, Inc., Germany). All samples had an RNA quality score greater than 8.0 prior to submission for sequencing (100-bp pair end reads) using the Illumina HiSeq2500 platform at the Research Technology Support Facility at Michigan State University (East Lansing, Michigan, USA). The FastQC program (www.bioinformatics.babraham.ac.uk/projects/fastqc/) was used to assess the quality of sequencing reads for the per base quality scores ranging from 30 to 40.

### Differential expression analysis and transcriptome annotation

RNA-seq reads of three biological replicates for nonchilled, chilled, and late-pink buds were analyzed. Two technical replicates were sequenced for each biological replicate and were combined together for analysis. The paired reads, two sets for each biological replicate, were aligned to the transcriptome reference Reftrinity developed for ‘Legacy’ [17] and the abundance of each read was estimated using the Trinity command “align_and_estimate_abundance.pl”. The Trinity command “run_DE_analysis.pl --method edgeR” was used for differential expression analysis. The DE transcripts with false discovery rate (FDR) values below 0.05 were used for further analyses. Comparison of transcriptome in chilled to nonchilled flower buds resulted in DE transcripts/genes in chilled flower buds. Comparison of transcriptome in late-pink buds to chilled flower buds resulted in DE transcripts/genes in late-pink buds. DE transcripts in chilled buds were annotated using Trinotate_v2.0 (https://trinotate.github.io).

### Gene network construction

Annotated transcripts were imported to Cytoscape 3.5.0 under BiNGO’s default parameters with selected ontology file ‘GOSlim_Plants’ and selected organism *A. thaliana* [39, 40].

### Identification of the selected pathway genes

Representative protein sequences of selected genes of *A. thaliana* were downloaded from the TAIR server (https://www.arabidopsis.org/tools/bulk/sequences/index.jsp). The retrieved sequences were used to search the blueberry transcriptome reference (refTrinity) using the tblastn command of BLAST+. The resultant transcripts that show e-value lower than − 20 were used to screen the DE transcript list of nonchilled floral buds.

The blueberry floral genes identified in the previous study [17] were used to analyze flowering pathway genes*.* The pathway genes of major phytohormones (gibberellin [41], abscisic acid [42], cytokinin/Arabidopsis Responsive Regulator [43], indole-3-acetic acid [44], and ethylene [45]) in *A. thaliana* were retrieved from TAIR_10 server based on published gene identities (Additional file 3: Table S3). Additionally, sequences of *A. thaliana* MADS-box proteins were used to analyze blueberry MADS-box genes. Percentages of DE phyotohormone genes were calculated based either on the number of orthologues to *A. thaliana* genes or on the number of DE blueberry transcripts.

### RT-PCR of DE transcripts

Reliability of DE genes or transcripts identified through RNA-seq was evaluated through qRT-PCR analysis of six selected transcripts (Additional file 4: Table S4). These transcripts are from the representative DE genes in auxin, ethylene, cytokinin, and gibberellin pathways. They have high fold changes (> 2) and sequence specificity (based on alignment result of different isoforms) for PCR amplification. Eukaryotic translation initiation factor 3 subunit H was the internal control (Additional file 4: Table S4).

The same RNA samples used for RNA-sequencing, including samples of three biological replicates, were used for cDNA preparation. Reverse transcription of RNA to cDNA was performed using SuperScript II reverse transcriptase (Invitrogen, Carlsbad, CA, USA). The resulting cDNA of one micro gram of RNA was diluted (volume 1: 4) in water and a 1 μl/sample (25 ng) was used for PCR reactions.

Integrated DNA Technologies, Inc. (https://www.idtdna.com/Primerquest/Home/Index) provided the online tool for primer design and synthesized the primers (Additional file 4: Table S4). Three qRT-PCR analyses were performed on an Agilent Technologies Stratagene Mx3005P (Agilent Technologies, Santa Clara, CA) using the SYBR Green system (Life Technologies, Carlsbad, CA). In each 25 μl reaction mixture, 25 ng of cDNA, 200 nM of primers, and 12.5 μl of 2× SYBR Green master mix were included. The reaction conditions for all primer pairs were 95 °C for 10 min, 40 cycles of 30 s at 95 °C, 60 s at 60 °C and 60 s at 72 °C, and followed by one cycle of 60 s at 95 °C, 30 s at 55 °C and 30 s at 95 °C. The specificity of the amplification reaction for each primer pair was determined by the melting curve. Transcript levels within samples were normalized to the eukaryotic translation initiation factor 3 subunit H. Fold changes were calculated by -∆∆Ct = − [(Ct_GOI_ – Ct_nom_) _tissue 1_ – (Ct_GOI_ – Ct_nom_) _tissue 2_] (*n* = 3).

## Additional files

Additional file 1: **Table S2.** DE MADS-box genes in chilled flower buds (CB) [vs nonchilled flower buds (NB)] and late-pink buds (LPB) (vs CB) in ‘Legacy’. LogFC for chilled buds: Log_2_(CB/NB). LogFC for late-pink buds: Log_2_(LPB/CB). Except #N/A (no differential expression), all the rest are DE genes. (XLSX 23 kb)

Additional file 2: **Table S1.** DE floral genes in chilled flower buds (CB) [vs nonchilled flower buds (NB)] and late-pink buds (LPB) (vs CB) in ‘Legacy’. LogFC for chilled buds: Log_2_(CB/NB). LogFC for late-pink buds: Log_2_(LPB/CB). #N/A: no differential expression. (XLSX 140 kb)

Additional file 3: **Table S3.** DE phytohormones in chilled flower buds(CB) [vs nonchilled flower buds (NB)] and late-pink buds (LPB) (vs CB) in ‘Legacy’. LogFC for chilled buds: Log_2_(CB/NB). LogFC for late-pink buds: Log_2_(LPB/CB). Except #N/A (no differential expression), all the rest are DE genes. (XLSX 401 kb)

Additional file 4: **Table S4.** Primers used in this study. (DOCX 54 kb)

## Abbreviations

ABA: abscisic acid
DE: Differentially expressed
FDR: False discovery rate
GA: gibberellin
GO: Gene ontology
IAA: indole-3-acetic acid
qRT-PCR: Quantitative reverse transcriptase polymerase chain reaction
