# Supplementary material for: Comparative transcriptome analysis of nonchilled, chilled, and late-pink bud reveals flowering pathway genes involved in chilling-mediated flowering in blueberry
Source: BMC Plant Biol. 2018 May 31;18:98. doi: 10.1186/s12870-018-1311-8 (PMC5984463; doi:10.1186/s12870-018-1311-8)
Supplement: Supplementary file 4 — Table S4. Primers used in this study. (DOCX 54 kb) [file 12870_2018_1311_MOESM4_ESM.docx]

**Table S4** Primers used in this study.

| Oligonucleotide name | Sequence (5’ to 3’) | Comments |
| --- | --- | --- |
| VcTFL1_Fwd | GTTGGAAGGGTGATAGGAGATG | c22179_g1_i1 |
| VcTFL1_Rev | GCCTGGAATGTCGGTGATTA | c22179_g1_i1 |
| VcARP6_Fwd | TGGTAGGTATGGGTCAGAAAGA | C49456_g2_i2 |
| VcARP6_Rev | GCTGTGGTCACAAAGGAGTAG | C49456_g2_i2 |
| VcFD_Fwd | CGGAGTCGGAAGTCAGAAATAC | c75407_g1_i2 |
| VcFD_Rev | GAGTGTCTCTTGGGAAGTTGAG | c75407_g1_i2 |
| Eukaryotic translation initiation factor 3 subunit H FWD | GAGAGATTCAGATGCCCAGAAG | C94438_g3_i2: VcEIF |
| Eukaryotic translation initiation factor 3 subunit H REV | GGACAATGGATGGACCAGATT | C94438_g3_i2: VcEIF |
